# East Texas Medico-Chirurgical Society

**Published:** 1901-06

**Authors:** Sam R. Burroughs, E. E. Guinn

**Affiliations:** President; Office of the Secretary, Rusk, Texas; Secretary; Office of the Secretary, Rusk, Texas


### East Texas Medico-Chirurgical Society.

*Reported officially for the Texas Medical Journal.*

OFFICE OF THE SECRETARY, RUSK, TEXAS, June 1, 1901.

The East Texas Medico-Chirurgical society met in the Komus Klub rooms at Palestine, May 30, 1901, with about forty-five members present. The meeting was called to order by Dr. A. L. Hathcock, chairman of the Committee on Arrangements, after which Rev. Mr. Campbell, of Palestine, invoked divine blessings upon the society.

Hon. C. M. Kay in a beautiful and impressive manner extended to the visiting physicians the hospitality of the thrifty little city.

The President, Dr. Sam R. Burroughs [The Sage of Buffalo.—ED.], in his usual beautiful and easy manner responded.

The roll was called, minutes of the last meeting read and approved, and seven new members were received into the society, viz.: Drs. J. M. Crawford, of Alto; T. J. Bell, of Tyler; W. B. Pullen, of Jacksonville; W. B. Collins, of Lovelady; J. L. Hall, of Crockett; B. F. Chambers, of Palestine, and L. Shumaker, of Palestine.

Section on General Medicine was taken up and Dr. J. H. Evans, of Palestine, read an interesting paper on "Pneumonia and Its Treatment."

Dr. J. H. Paxton presented a paper upon "Hemoglobinuria." These subjects were discussed thoroughly by most of the members present.

At 8 p. m. Dr. Burroughs delivered the address in memory of Dr. J. H. McDaniel, after which the Society adjourned to the Nolen Hotel, where the Committee on Arrangements had provided a magnificent feast, composed of all the delicacies known to culinary art that man's appetite could desire.

When a reasonable quantity of champagne had been consumed, that honored old warrior, the distinguished "Daniel, of the 'Red-Back,'" was called for, and it being his nature to (at all times and in all places) make his fellow-man feel glad that he still liveth, broke forth with characteristic humor and eloquence, such as few men possess. (We always feel glad when the Doctor is present, and all others do also.) Dr. Daniel was made an honorary member.

The Hon. Ben F. Rogers, ex-State Senator, of Palestine, followed Dr. Daniel, and the manner in which he discussed the medical profession was certainly calculated to make every doctor present feel like he was surely one of the essential creatures of today. Long after both hands of the clock pointed straight up, we retired to dream of the memories of the occasion.

At 9 o'clock a. m. of the 31st the meeting was called to order by the President, and executive business was taken up. Secretary and Treasurer's reports were received, showing a healthy cash balance on hand.

Under this head, the election of officers was entered into with the following results:

Dr. W. C. Lipscomb, of Crockett, President.

Dr. A. L. Hathcock, of Palestine, First Vice-President.

Dr. E. P. Murdock, of Oakwoods, Second Vice-President.

Dr. J. M. Colly, of Palestine, Treasurer.

Dr. E. E. Guinn, of Rusk, Secretary.

The following were chosen as Board of Censors: Drs. T. J. Bell, of Tyler; J. H. Paxton, of Elkhart; Z. J. Spruiell, of Jewett; J. N. Gee, of Bethel; W. B. Pullen, of Jacksonville.

The following were appointed as chairmen of Sections: On General Medicine, Dr. Jno. H. Joyce, of Buffalo; General Surgery, Dr. E. W. Link, of Palestine; Gynecology and Obstetrics, Dr. T. J. Bell, of Tyler; Diseases of Children and Therapeutics, Dr. L. Merriwether, of Grapeland.

Arrangement Committee: Drs. A. L. Hathcock, E. W. Link and E. B. Parsons, of Palestine.

Dr. W. B. Collins, of Lovelady, offered the following resolution, which was adopted:

"Resolved, That the East Texas Medico-Chirurgical Society unanimously thank Dr. R. H. Harrison, of Columbus, for his untiring work in behalf of the advancement of the profession of medicine in Texas, and cheerfully record his name as an honorary member of this Society; and as a further evidence of our appreciation, that this Society present him with a nice gold-headed cane, appropriately inscribed." The President appointed Drs. W. B. Collins, L. Merriwether and John Colly as a committee to select and present to Dr. Harrison the cane.

The following resolutions were also adopted:

Resolved, That this Society heartily endorse the action of the State Medical Association in its selection of the eighteen members of our profession, from which the Governor may appoint the members of the State Medical Examining Board; and especially do we endorse the selection of Dr. J. H. Evans, of the Second District, and our President, Dr. Sam R. Burroughs, who was selected from the State at large.

Resolutions of thanks were tendered the retiring officers of the Society, the Komus Klub, Hon. C. M. Kay, and the efficient Arrangement Committee.

An impressive feature of the day was a memorial delivered by Dr. Sam R. Burroughs, a tribute to the memory of the late Dr. J. H. McDaniel, of Centerville, Texas, followed by Dr. J. H. Joyce.

Mrs. S. Terry, State agent for the "Red-Back," honored our meeting with her presence.

Section work was taken up, and the following papers were presented: "Membranous Croup," by Dr. Z. J. Spruell; "Appendicitis," by Dr. A. L. Hathcock; "Chronic Gonorrhea," by Dr. R. H. McCloud; "Gall Stones," by Dr. W. I. M. Smith; "Drainage in Surgery," by Dr. F. C. Woodard; "Puerperal Eclampsia," by Dr. E. B. Parsons; "Pelvis Peritonitis," by Dr. W. C. Lipscomb; "Antitoxin," by Dr. M. D. McCarty; "Summer Diarrhea," by Dr. E. E. Guinn; "Intubation vs. Tracheotomy," by Dr. G. R. Howard.

The Society adjourned to meet again on the 28th of November, 1901, in the city of Palestine.

SAM R. BURROUGHS,

President.

E. E. GUINN,

Secretary.

